# Aggressive Multimodal Approach for Anaplastic Thyroid Cancer and Long-Term Survival

**DOI:** 10.1155/2013/783862

**Published:** 2013-02-27

**Authors:** Nasir Hussain, Usman Mustafa, Su Hyeon Jung, Alan D. Gilman

**Affiliations:** ^1^Department of Internal Medicine, Saint Joseph Hospital, Presence Health, 2900 North Lake Shore Drive, Chicago, IL 60657, USA; ^2^Department of Hematology and Oncology, Saint Joseph Hospital, Chicago, IL, USA

## Abstract

Anaplastic thyroid cancer (ATC) comprises 1-2% of all thyroid cancers and is one of the most aggressive cancers with a median survival rate of around four months. The average 5-year survival rate has been reported to be around 3.6%. In this paper, we have discussed management and prognostic variables of a patient with ATC who has survived for more than 5 years. A 59-year-old female was referred to our facility for an elective thyroid and parathyroidectomy for concerns of thyroid papillary cancer and hyperparathyroidism. At the time of surgery, the tumor mass had invaded the muscular layer of esophagus; radicle thyroidectomy parathyroidectomy along with removal of muscle layer of esophagus was performed, and diagnosis of ATC was made. The patient was treated with chemoradiation with a good treatment response and no recurrence of tumor for two and a half years until PET/CT followed by wedge biopsy of lung confirmed ATC recurrence. The patient was treated with another course of radiation treatment with a good treatment response. Since then, the patient has been following in our outpatient oncology clinic and has no evidence of tumor recurrence. Aggressive multimodal approach of combining radicle surgery with chemoradiation treatment in select patients of ATC with no distant metastasis helps improve prognosis.

## 1. Introduction 

Anaplastic thyroid cancer (ATC) comprises 1-2% of all thyroid cancers and is one of the most aggressive cancers with a median survival rate of a few months [1]. ATC contributes up to 14–50% of the annual mortality due to thyroid cancer [2]. The average 5-year survival rate for ATC is around 3.6% [3]. In this paper, we describe treatment course of a patient who has survived for more than five years following the diagnosis of ATC. We have also provided concise review of the prognostic variables that affect the outcome in cases of ATC.

## 2. Case Presentation

**Figure 1:**
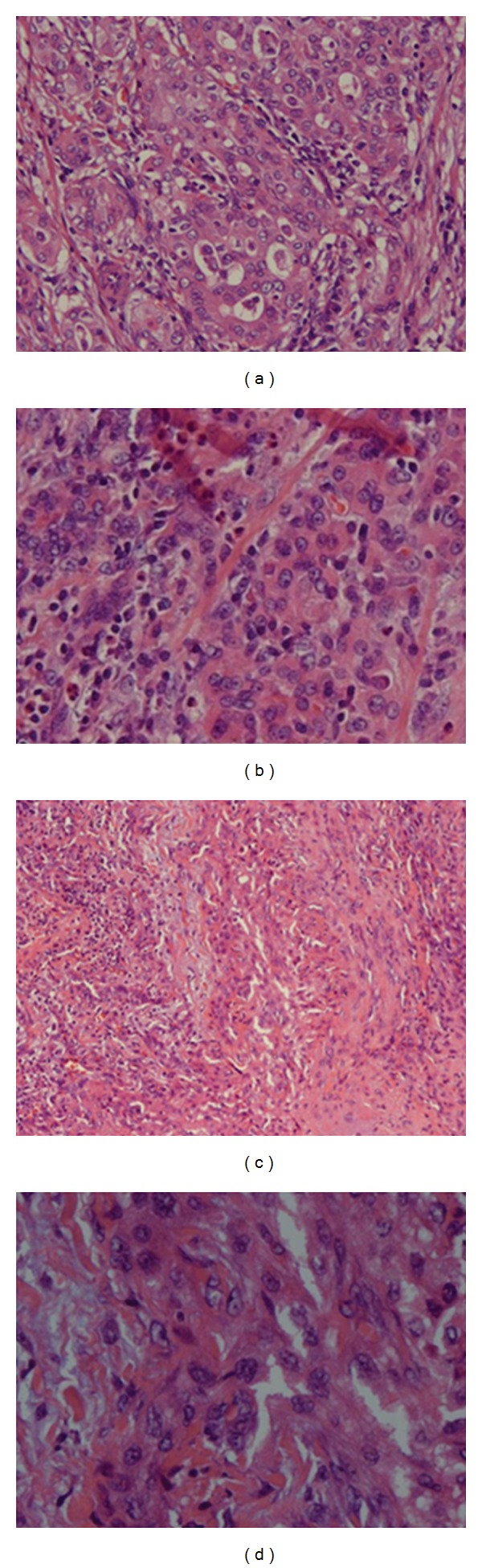
(a, b, d) Poorly differentiated ATC, eosinophilic cytoplasm of neoplastic cells, irregular nuclei with coarse chromatin, and prominent nucleoli; (c) recurrent ATC involving upper lobe of the lung.

A 59-year-old female with a past medical history of breast cancer (status after radiation therapy (RT) and a subsequent mastectomy for tumor recurrence), osteoporosis, and hypercalcemia was referred for elective thyroid and parathyroidectomy. The patient had a thyroid lump for months which on imaging (PET CT scan) and ultrasound guided biopsy was found to be a papillary cancer with no distant metastasis (two weeks prior to the presentation). A parathyroid localization scan one day prior to the presentation was suggestive of parathyroid adenomas. At the time of presentation, vital signs, physical examination, and basic diagnostic lab test including complete blood count, comprehensive metabolic panel, and thyroid function tests were within normal limits except for a serum calcium level of 10.8 mg/dL (8.4–10.5 mg/dL). On December 20, 2007, the patient underwent a radicle surgical procedure where total thyroidectomy, parathyroidectomy and thymectomy were done. Muscular layer of esophagus was resected to ensure complete removal of the tumor. Esophageal repair and lymph nodes' dissection were performed and laryngeal nerves were preserved. Diagnosis of parathyroid hyperplasia and poorly differentiated ATC (Figure 1) with few nests of well-differentiated papillary cancer in a background of multinodular goiter with no involvements of regional lymph nodes was made. Histological examination of the biopsy specimen revealed that ATC approached within 1 mm of resection margins at several locations and was poorly differentiated with prominent eosinophilic cytoplasm of neoplastic cells, irregular nuclei with coarse chromatin, and prominent nucleoli. The biopsy specimen stained positive for cytokeratin AE1/AE3, cytokeratin CK5/6, and was negative for thyroid transcription factor-1, thyroglobulin, vimentin, smooth muscle actin, leucocyte common antigen, and carcinoembryonic antigen. During postoperative course, the patient did well and was discharged with instructions to continue with levothyroxine and to follow up in an outpatient oncology clinic. Subsequently, the patient was started on carboplatin, paclitaxel with concomitant RT. The patient tolerated the RT well except for some odynophagia necessitating a break of one week during the course of RT treatment. RT was delivered in daily fractions for a total of 36 days using three-dimensional conformal radiation therapy (3D-CRT) followed by intensity-modulated radiation therapy (IMRT) (total dose, 60 Gray). Chemotherapy was given in seven cycles for four months. The patient was closely monitored with frequent PET/CT scans, thyroid function tests, thyroglobulin, and thyroglobulin antibodies levels. In May 2010, PET/CT scan identified a hot 1.1 cm nodule in the right pulmonary apex which on wedge biopsy was found to be a metastatic ATC. The metastatic focus of the lung also stained positive for cytokeratin AE1/AE3, cytokeratin CK5/6, and was negative for thyroid transcription factor-1, thyroglobulin, vimentin, smooth muscle actin, leucocyte common antigen, and carcinoembryonic antigen, same as the original ATC tumor. The patient was given another course of RT for 46 consecutive days using 3D-CRT (total dose, 66 Gray), which patient tolerated well. Since then, the patient has been in a good state of health and has been regularly following in our oncology clinic without any evidence of tumor recurrence. 

## 3. Discussion

ATC is three times more common in females as compared to males with a peak incidence seen during the sixth or seventh decade of life [1]. ATC carries a median survival rate of approximately four months [4] and is considered metastatic at the time of diagnosis by the American Joint Committee on Cancer. Preexisting goiter or a family history of goiter [5] and previously undetected long standing thyroid cancers have been identified as risk factors for ATC. Our patient did have an evidence of multinodular goiter on the histological examination of resected thyroid gland.

Incidence of ATC has declined with the use of iodine [6] and with better immunohistochemical techniques for identification of ATC [1]. 

ATC commonly presents as a central neck mass and may or may not have an associated dysphagia, voice changes, or stridor [1]. Regional lymph node swelling and neck pain along with usual systemic symptoms of anorexia and weight loss are commonly present at the time of diagnosis [1]. Nearly half of the ATC patients have pulmonary metastasis at the time of diagnosis with another quarter developing pulmonary metastasis during the course of the disease [1]. Lungs, bone, and brain are the most common sites of metastasis [7]. 

Favorable prognostic variables for ATC identified so far are age less than 60 years, tumor size less than 6-7 cm, and treatment with radicle surgery [1]. The use of RT after surgery in cases of extracapsular extension of tumor with no distant metastasis, has been found to be useful [1]. The role of gender as outcome variable for ATC is controversial [1, 8]. Presence of acute symptom, leukocytosis, tumor invasion of thyroid capsule, tumor residue after surgery, lymph node metastasis, distant metastasis and a lack of multimodal treatment have been shown to be determinants of poor prognosis [1, 8]. In our case, the patient had multiple favorable prognostic variables which could have contributed to her long-term survival. The patient had no acute symptoms at the time of presentation, had no leukocytosis, and was less than 60 years of age, and tumor was less than 6 cm in size with extracapsular extension and esophageal involvement but with no distant metastasis or lymph node involvement. There was no evidence of any residual tumor after the radicle surgery as followup PET/CT scans were within normal limit. 

Treatment modalities available for ATC are chemotherapy, radiation treatment, and surgery; combined approach yields better survival results than any of the modalities alone [1]. Both modalities of RT (IMRT and 3D-CRT) are considered to be equally effective in the treatment of ATC [9]. There is no consensus on which antineoplastic regimen to consider in setting of ATC. Doxorubicin [10], cisplatin [11], paclitaxel [12], carboplatin [13], and valproic acid [14] alone or in combination have been commonly used along with other treatment modalities with variable success rates. In our patient, radicle surgery combined with radiation and chemotherapy achieved aggressive local control of the tumor. 

New antitumor therapies like tyrosine kinase inhibitors (imatinib mesylate) [15], VEGF-R inhibitors (axitinib), BRAF inhibitors [16] (sorafenib), EGFR monoclonal antibody (cetuximab) [17], and vascular disrupting agents like fosbretabulin [18] are being considered for the treatment of ATC. Multiple other agents are in the midst of clinical evaluation [1]. 

Our case underscores that aggressive multimodal approach may improve prognosis for ATC in select patients. Future studies are needed to better predict outcome variables and to determine optimal multimodal approach for the treatment of ATC.
